# Etymologia: dengue

**DOI:** 10.3201/eid1206.ET1206

**Published:** 2006-06

**Keywords:** dengue, flavivirus, Aedes mosquitos, water poison

## [den′gē]

An acute, self-limited disease characterized by fever, headache, myalgia, and rash caused by any of 4 related but distinct viruses of the genus *Flavivirus* and spread by *Aedes* mosquitos. Dengue (a Spanish homonym for the Swahili *ki denga pepo*, which describes a sudden, cramplike seizure caused by an evil spirit) is believed to have been first recorded in a Chinese medical encyclopedia from the Chin Dynasty (265–420 AD). The Chinese called dengue “water poison” and knew that it was somehow associated with flying insects.

Sources: Dorland’s illustrated medical dictionary. 30th ed. Philadelphia: Saunders; 2003; Gubler DJ. Dengue and dengue hemorrhagic fever. Clin Microbiol Rev. 1998;11:480–96; and Halstead SB. Dengue hemorrhagic fever—a public health problem and a field for research. Bull World Health Organ. 1980;58:1–21.

